# The development of a new service for the management of Non-inflammatory Musculoskeletal Pain

**DOI:** 10.1186/1546-0096-9-S1-O36

**Published:** 2011-09-14

**Authors:** S Bhagat, C Bernie, A Morgan, C Pilkington, SM Maillard

## Introduction

Great Ormond Street Hospital Rheumatology Department recognised that 50% of the referrals that were received were for young people with non-inflammatory musculoskeletal pain (NIMP) but that the service was not able to provide adequate treatment after diagnosis. A business plan was proposed to the hospital management board and a new service was developed and reviewed.

## Methods

The business plan was developed by senior multidisciplinary members of the Rheumatology team. The new service was set up and then specific outcome measures were followed after 1 year to monitor the success and effectiveness of the treatments.

## Results

The service was funded to employ a full time Physiotherapist (PT), occupational therapist (OT), psychologist (Psych) and administrator. Outpatient appointments were established and patients were placed into the new service. The initial appointment was in a new-patient clinic and they were assessed by a Consultant paediatric Rheumatologist and a specialist physiotherapist. This initial assessment ensured that there was no other diagnosis other than a non-inflammatory condition such as hypermobility syndrome; the patient was then referred to PT, OT and Psych as appropriate. The treatments included individualised home muscle strength training programme, life style advice, group patient and parent education programmes and individualised pain management interventions. Specific Outcome measures (OCM) were used to monitor the success of the treatments provided. The most significant OCM’s were:

• School attendance which increased from 75% missing at least 1 day a week due to pain to only 7%.

• Loss of muscle strength in 98% of children which was resolved in 100% of those who completed the programme given.

• Participation in PE also increased from 25% participating to 75% joining in sport at school.

• The cost of providing the staff and facilities was only 30% of the revenue the service brought into the hospital.

## Conclusion

The establishment of a dedicated service to the management of NIMPS has been shown to be both clinically and cost effective and has worked extremely well as an AHP led service enabling the medical staff to focus upon inflammatory diseases.

